# Changes in the intestinal expression of drug metabolism-related genes in a piglet model of parenteral nutrition

**DOI:** 10.1186/s12986-022-00654-8

**Published:** 2022-03-09

**Authors:** Li-Na Dai, Yu-Ling Zhao, Lu Jiang, Jun-Kai Yan

**Affiliations:** 1 Department of Pediatric Surgery, Xin Hua Hospital, School of Medicine, Shanghai Jiao Tong University, 1665 Kongjiang Rd, Shanghai, 200092 China; 2 Shanghai Key Laboratory of Pediatric Gastroenterology and Nutrition, Shanghai Institute for Pediatric Research, Shanghai, China; 3 Department of Urology, Shanghai Children’s Medical Center Affiliated to Shanghai Jiao Tong University School of Medicine, Shanghai, China

**Keywords:** Parenteral nutrition, Drug metabolism-related genes, Proteomics, Fibroblast growth factor 19, Ileum organoids

## Abstract

**Background:**

Parenteral nutrition (PN) may serve as a nutritional supportive therapy accompanied by oral medication, but the effect of PN on intestinal expression of drug metabolism-related genes remains unknown.

**Methods:**

Twelve Bama piglets receiving PN for 14 days were used as in vivo model. Changes in intestinal drug metabolism-related genes were examined by proteomic analysis. Serum levels of fibroblast growth factor 19 (FGF19) were determined by ELISA, and the effect of FGF19 on the expression of drug metabolism-related genes was examined using murine ileum organoids.

**Results:**

A total of 1063 differentially expressed proteins were identified in PN group. Of note, two drug transporters (Abcb1 and Abcc2) were significantly decreased in PN group, along with two glutathione-related drug-metabolizing enzymes, glutathione peroxidase (Gpx2) and glutathione S-transferase (Gsta1). Serum FGF19 levels were dramatically reduced in PN group. Treatment with recombinant FGF19 in vitro dose-dependently up-regulated the expression of Abcb1, Abcc2, Gpx2 and Gsta1 in organoids.

**Conclusion:**

Our data indicated that intestinal drug metabolism-related genes were significantly dysregulated under PN, and some of the changed genes were attributed to gut-derived FGF19.

## Introduction

Parenteral nutrition (PN) is a vital therapy for patients with impaired gut function who cannot tolerate enteral nutrition (EN), including severe inflammatory bowel diseases, short bowel syndrome and chronic intestinal pseudo-obstruction [1–3]. As a nutritional supportive therapy that may be accompanied by oral medication, we assumed that micro-environment of drug metabolism in those patients receiving PN may be different from that in those patients receiving EN. For instance, a number of studies using both PN animal models and human samples reported that genes involved in drug metabolism changed significantly in liver, suggesting that the process of drug metabolism might be disturbed by liver injury [4, 5]. Given that gut also plays a pivotal role in drug metabolism, especially the absorption of oral drugs, and that intestinal homeostasis is dramatically disrupted during PN, we assumed that intestinal expression of drug metabolism-related genes might be dysregulated, leading to impaired metabolic pathways in gut beyond liver.

Proteomic profiling is a large-scale comprehensive investigation of proteins, including information on protein abundance and modification, and their interacting networks. To the best of our knowledge, neither transcriptomic nor proteomic investigation in gut tissues have been conducted under PN background till date. Therefore, it is necessary to clarify the effect of PN on the proteome characteristics of small intestine, which may affect the pharmacokinetic profile of oral drugs. ATP-binding cassette (ABC) transporters have been reported to play a major role in protecting against dietary toxicants as well as limiting the oral bioavailability of drugs of therapeutic use [6], most notably ATP binding cassette subfamily B member 1 (Abcb1, also known as multidrug resistance protein 1) and ATP binding cassette subfamily C member 2 (Abcc2, also known as multidrug resistance-associated protein 2). Additionally, glutathione-related drug-metabolizing enzymes have been reported to play a pivotal role in biotransformation to protect against dietary oxidants and endogenously produced reactive oxygen species (ROS) in the gastrointestinal tract [7], including glutathione-s-transferases (GSTs) and glutathione peroxidases (GPXs). On the other hand, previous studies have demonstrated that gut-derived peptide hormone fibroblast growth factor 19 (FGF19), a postprandial hormone induced by Farnesoid X receptor (FXR) upon activation by bile acids, plays a fundamental role in the modulation of various metabolic processes [8]; and more importantly, reduced levels of circulating FGF19 have been implicated in the severe complications of PN use, notably PN-associated liver disease (PNALD) [9]. We hypothesized that intestinal drug metabolism-related genes might be significantly dysregulated under PN, and some of the changed genes might be attributed to gut-derived FGF19. This present study provided new insights into changes in intestinal drug metabolism-related genes under PN, and preliminary evidence showing the association of FGF19 with drug metabolism.

## Material and methods

### Reagents

Cell culture reagents for ileum organoids were purchased from STEMCELL (#06005, USA). Recombinant human FGF19 protein was purchased from Abcam (#ab200246, USA). GSH/GSSG kit was purchased from Beyotime (#S0053, China). All other chemicals were purchased from Sigma (China).

### PN piglet model

Twelve 2-day-old Guangxi Bama minipigs (0.7–1 kg) were housed in a heat- and light-controlled room with 12 h light/dark cycle, randomized to EN and PN group (n = 6/group). Surgery procedure and PN formula was identical to our previous work [10]. Briefly, BTPU-040 catheters (Instech, USA) were used for cannulation via external jugular vein. For PN group, the piglets were parenterally fed with PN solution containing 240 ml/kg/day of protein (13 g), lipid (5 g) and carbohydrate (25 g); Detailed formula shown in Table 1. PN piglets received 50% of the total requirement of the PN solution during the first 24 h. EN pigs were fed with milk powder containing soy protein and glucose, providing digestion energy of 4,050 kcal/kg and 48 g/kg in 240 mL of warm water. All animals were sacrificed at 14 days after surgery. This study was approved by Xin Hua Hospital Animal Use Committee (XHEC-F-2020–008).

**Table 1:** Components of parenteral nutrition solution

Component	Volume (ml)
50% dextrose	50
8.5% amino acids	153
20% MCT / LCT	25
10% sodium chloride	2.4
10% potassium chloride	2.4
Trace elements mix	0.5
Water-soluble vitamins mix	0.5
Fat-soluble vitamins mix	0.5
10% calcium gluconate	4.6
Sodium glycerophophate	4.6
magnesium sulfate	0.33
Total	243.83

*LCT* long-chain triglyceride; *MCT* medium-chain triglyceride

### Sample collection

Ileal tissues were isolated about 10 cm proximal to the ileocecal valve. The segments were divided into several parts (1–2 cm each) for intestinal morphology assessment, proteome analysis, RNA isolation and glutathione measurement. All the samples were stored at − 80 °C until further use.

### Intestinal morphology assessment

Formalin-fixed and paraffin-embedded tissue Sects. (5 μm) were stained using standard immunohistochemistry procedures. Villus height and crypt depth were measured in 20+ well oriented, full-length crypt/villus units per specimen and averaged. Data were analyzed with LAS AF LITE image processing software (Leica, Germany).

## Proteome analysis

Ileum protein extracts were generated by homogenizing 20 mg tissue in 200 μL lysis buffer (4% SDS, 100 mM DTT, 150 mM Tris–HCl pH 8.0), followed by tandem mass tag (TMT)-labelling and proteome analysis using LC–MS/MS. TMT-labelled peptides were loaded onto a the C18-reversed phase column (12 cm long, 75 μm ID, 3 μm) in buffer A (2% acetonitrile and 0.1% Formic acid) and separated with a linear gradient of buffer B (90% acetonitrile and 0.1% Formic acid) at a flow rate of 300 nL/min over 60 min as follows: 0–2 min, linear gradient from 2 to 5% buffer B; 2–42 min, linear gradient from 5 to 20% buffer B; 42–50 min, linear gradient from 20 to 35% buffer B; 50–52 min, linear gradient from 35 to 90% buffer B; 52–60 min, buffer B maintained at 90%. MS data was acquired using a data-dependent top 15 method dynamically choosing the most abundant precursor ions from the survey scan (300–1800 m/z) for high-energy collisional dissociations (HCD) fragmentation.Dynamic exclusion duration was 30 s. Survey scans were acquired at a resolution of 70,000 at m/z 200 and resolution for HCD spectra was set to 17,500 at m/z 200. Normalized collision energy was 30 eV. Normalized collision energy was 30.

### Database searches and bioinformatics analysis

The resulting LC–MS/MS raw files were imported into MaxQuant software (version 1.6.0.16) for data interpretation and protein identification against the database Uniprot_Hordeum-vulgare_201747-20180125 (downloaded on 25/01/2018, and including 201747 protein sequences). Differentially expressed proteins (DEPs) were defined in the TMT experiment according to the following criteria: unique peptides ≥ 1, *p*-value < 0.05, fold change > 1.2 or < 0.83 [4]. Bioinformatics analysis were then performed, including Gene ontology (GO)-term classification (david.abcc.ncifcrf.gov), and Protein–protein interaction (PPI) networks (v10, string-db.org).

### Quantitative real-time PCR (qPCR)

Total RNA was extracted using Trizol (life technology, USA), and cDNA was synthesized with RNA isolation plus kit (Takara, Japan) according to the manufacturer’s protocol. Quantitative real-time PCR was performed using PIKO96 (Thermo, Germany). Primers used in this study are listed in Table 2.

**Table 2:** Primers for quantitative RT-PCR analysis

Gene	Forward	Reverse	Product size (bp)
sAbcb1	TGTTCAACTACCCCACTCG	TTTAATCTCCCTGCCGTCA	185
sAbcc2	AAAAAGCGTGCGTAAAATA	ACAGAACGAACCTGGAAAA	429
sGsta1	AACAACAGCTTACAAACCC	ATACACTCCATTCTGCCTC	155
sGstm3	CTTTCCTAACCTGCCCTAT	TTTCTTCTTCAGTCTCCCC	113
sGstt1	CCATCTACATCTTCGCCAA	GCAGGGTCACATCCAACTC	387
sGpx2	ACGGGGAGAAGGTAGATTT	CGTTGAGTTGGGTGAAGTC	105
sGpx8	CCTTTCTGACTTTCCGTTA	TTCCACCTTGGTTCCTTCT	325
sHprt1	CCATCACATCGTAGCCCTCT	GCTTGCAACCTTGACCATCT	310
mAbcb1a	AACTGCCCCACCAATTTGAC	TCTAGCCTTATCCAGTGCGG	188
mAbcb1b	ACTCGGGAGCAGAAGTTTGA	CATGAGTTGTTGTGCCACCA	177
mAbcc2	ATGGGACCGACAATTCACCT	CCCGGCAAATCTGTTCACAA	227
mGsta1	GCCAGGACTCTCACTAGACC	CATGGGCACTTGGTCAAACA	210
mGstm3	GACTCACTCCATCCGCTTG	TGGCTTCTGCTTCTCAAAA	337
mGstt1	CCAGTCTTTGAAGGGCATCC	ATGGTGTGAGGAGTGAGGTG	280
mGpx2	GACAAGCTGCCCTACCCTTA	TCAGGCTCGATGTTGATGGT	182
mGpx8	AGTCAGGCCAGTACAACAGG	AAGAATGGAGATGGAGAAG	285
mActb	CCTCTATGCCAACACAGTGC	GCCTTCACCGTTCCAGTTTT	339

“s” and “m” refer to pig (*Sus scrofa*) and mouse (*Mus musculus*) genes, respectively

### Glutathione measurement

Oxidized glutathione (GSSG) and reduced glutathione (GSH) were measured using a glutathione kit (Beyotime, China). Briefly, 10 mg ileum tissue obtained from 10 cm proximal to the ileocecal valve was homogenated in 0.5 ml cold GSH assay buffer, followed by centrifugation at 100,000 g for 10 min. GSH and GSSG levels were determined according to manufacturer’s instruction, and were normalized to tissue weight.

### FGF19 levels

Blood was taken via portal vein, and serum FGF19 levels in portal vein were determined using an ELISA kit according to manufacturer’s protocol (#ELP-FGF19, Raybiotech, USA).

### Culture and treatment of ileum organoids

Isolation and culture of primary organoids were conducted following the method described previously [11]. Briefly, primary crypts were released from the ileum mucosa of a C57BL wild-type mouse (8w, male). Isolated crypts were then mixed with Matrigel (Corning Inc, Corning, NY) and cultured in murine intestinal organoid growth medium (#06005, STEMCELL). For FGF19 treatment, organoids were treated with recombinant human FGF19 (20 and 50 ng/ml) at 1-day after seeding for 6 days. Morphological changes were examined under phasecontrast microscopy.

### Transport assay in organoids

For Abcb1 function assay, Rhodamine 123 (Rho 123) assay was performed as described previously [12]. Briefly, organoids were incubated with growth medium containing Rho 123 (10 μM) in the dark at 37 °C for 1 h. Afterwards, these organoids were washed five times with PBS and then incubated in culture medium to allow Rho 123 efflux in the dark at 37 °C for 1 h, with or without Verapamil (100 μM, a specific inhibitor for Abcb1). A 100 μl aliquot of sample was taken for the determination of Rho 123 content using a fluorescence spectrophotometer (SpectraMax, CA, USA) under ex/em = 485 nm/520 nm. Alternatively, organoids were also imaged using Leica DMI6000B fluorescence microscopy with LAS AF LITE image processing software (Leica, Germany).

### Statistical analysis

Data are expressed as means ± SD, statistically analyzed and plotted using GraphPad Prism 8.0 (GraphPad Software Inc., San Diego, CA, USA). Variables were analyzed either by Student’s t-test for two groups or ANOVA analysis for multiple groups, with statistical significance when *p* < 0.05.

## Results

### Proteome characteristics of ileum tissue from piglet model of PN

**Fig. 1:**
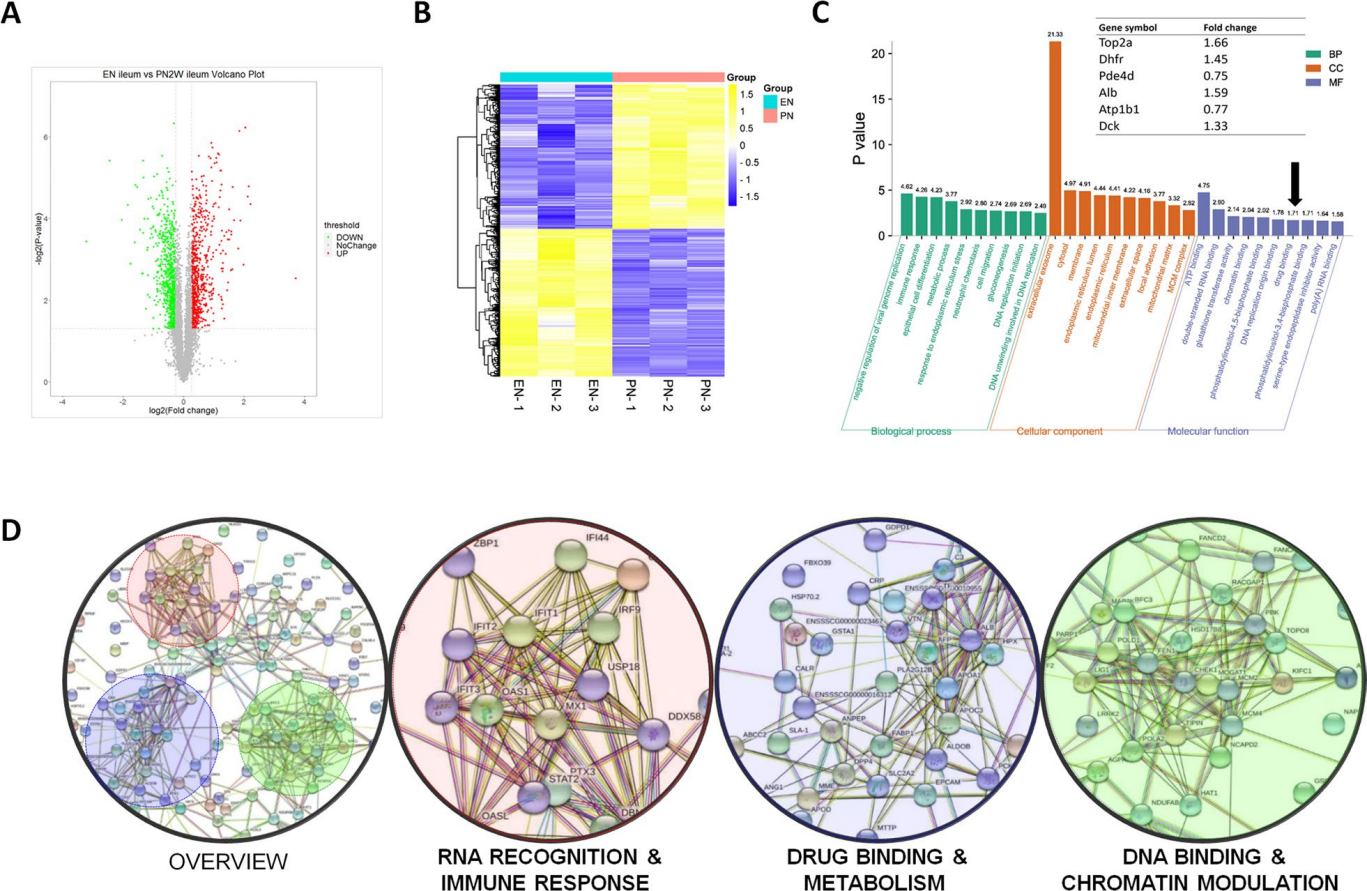
Proteome characteristics of ileum tissue from piglet model of PN. **A** Volcano plot depicting the protein changes induced by PN treatment. Plots are up-regulated (red) or down-regulated (green) proteins with fold change > 1.2 or < 0.83 (*p* < 0.05). **B** Heat map of differentially expressed proteins (DEPs). **C** Gene Ontology analysis of DEPs. *BP* biological process; *CC* Cellular component; *MF* molecular function **D** Protein–protein interaction of DEPs. Three key interactions were identified, including RNA recognition & immune response (red), drug binding & metabolism (blue) and DNA binding & chromatin modulation (n = 3/group)

In ileum tissues, a total of 40,682 peptides and 6534 proteins were identified by TMT analysis, and 1063 proteins were significantly changed in PN group (Fig. 1A), including 540 down-regulated proteins and 523 up-regulated proteins (Fig. 1B). In GO-term analysis, a number of DEPs involved in drug binding (MF #7/10) were identified by annotation clustering (Fig. 1C). PPI analysis revealed that interaction between DEPs could be roughly clustered to three fields (Fig. 1D), including RNA RECOGNITION & IMMUNE RESPONSE (red), DRUG BINDING & METABOLISM (blue) and DNA BINDING & CHROMATIN MODULATION (green).

### Effect of PN on ABC transporters and glutathione-related genes

**Fig. 2:**
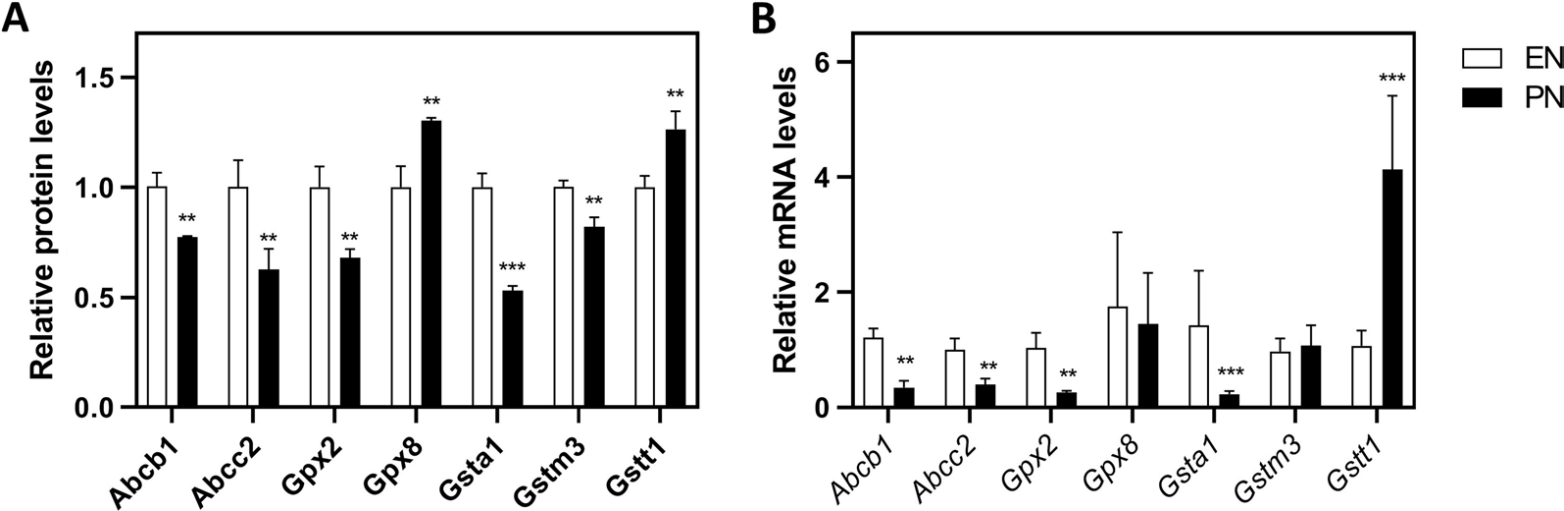
Changes in the intestinal expression of ABC transporters and Glutathione-related genes in vivo. **A** Relative protein levels determined by LC–MS. Ileum tissues were isolated from 14d-PN piglets. Data are presented as mean ± SD. **p < 0.01, ***p < 0.001 (n = 3/group). **B** Relative mRNA levels determined by qPCR using ∆∆Ct. Data are presented as mean ± SD. **p < 0.01, ***p < 0.001 (n = 6/group)

For ABC transporters, Abcb1 (0.77-fold vs EN) and Abcc2 (0.62-fold vs EN) significantly decreased in PN group. For glutathione peroxidases, Gpx2 significantly decreased in PN group (0.67-fold vs EN), while Gpx8 increased (1.3-fold vs EN). For glutathione transferases, Gsta1 (0.53-fold vs EN) and Gstm3 (0.81-fold vs EN) significantly decreased in PN group, while Gstt1 (1.26-fold vs EN) increased (Fig. 2A). Consistently, qPCR data indicated that mRNA changes basically mirrored protein changes except *Gpx8* and *Gstm3* (Fig. 2B).

### Effect of PN on glutathione levels in ileum

**Fig. 3:**
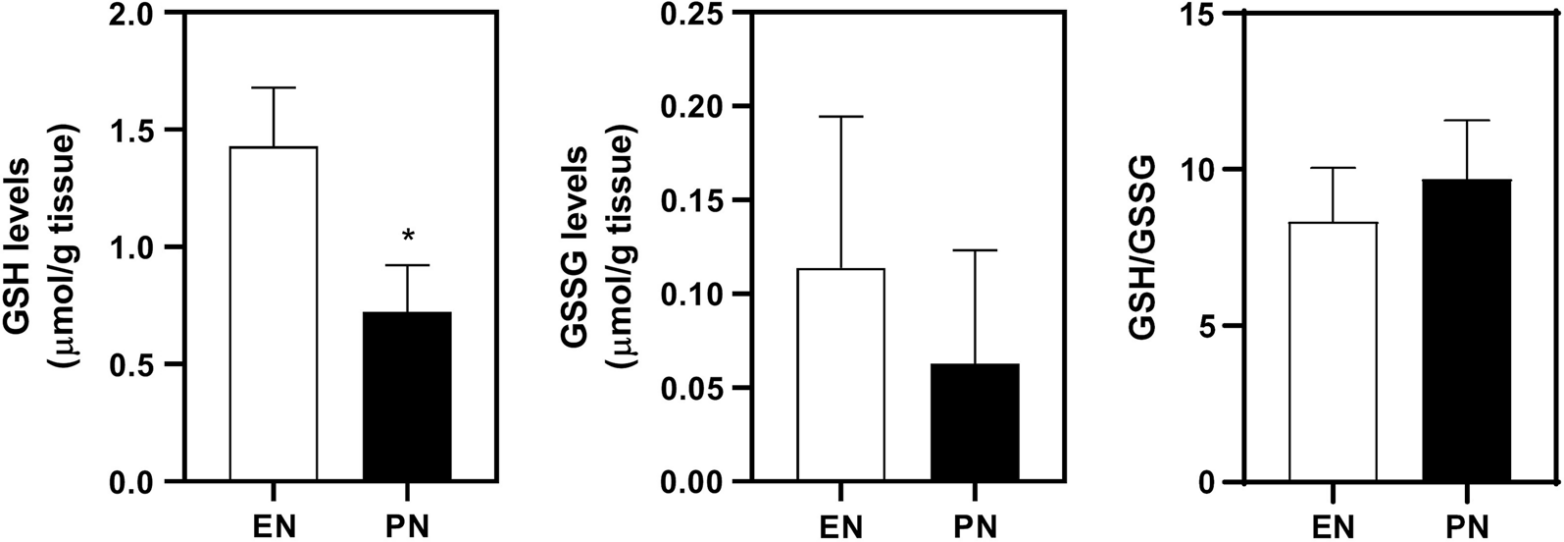
Total levels of GSH, GSSG and GSH/GSSG ratio in ileum tissue. Ileum tissues were isolated from 14d-PN piglets. Data are presented as mean ± SD. *p < 0.05 (n = 6/group)

In addition to glutathione-related genes, glutathione levels also changed significantly in PN group. As shown, GSH levels were 1.42 ± 0.20 μmol/g tissue and 0.72 ± 0.16 μmol/g tissue in EN and PN group (*p* < 0.05), respectively (Fig. 3A). However, GSSG levels were slightly reduced without statistical differences (Fig. 3B), and consequently there were no significant differences in GSH/GSSG ratio between groups (Fig. 3C).

### Changes in morphological features of ileum and FGF19 levels

**Fig. 4:**
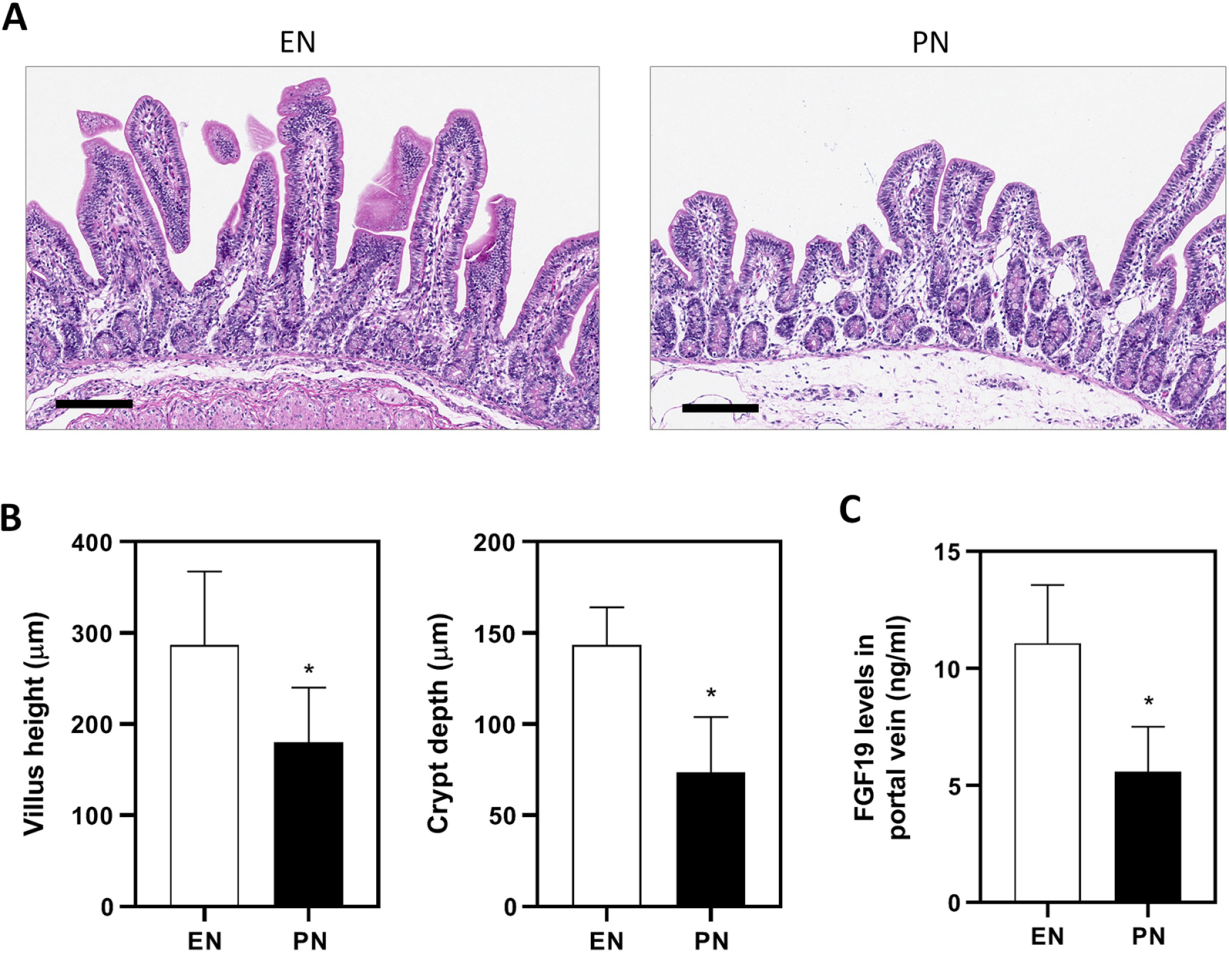
Effect of PN on ileum epithelium and serum FGF19 levels. **A** Representative images showing villus atrophy in the ileum epithelium. Scale bar = 100 μm. **B** Quantification of villus height and crypt depth in (**A**). Data are presented as mean ± SD. *p < 0.05 (n = 6/group). **C** Serum levels of FGF19 in portal vein. Data are presented as mean ± SD. *p < 0.05 (n = 6/group)

After 14d treatment, PN group exhibited significant villus atrophy in ileum tissues (Fig. 4A), characterized by decreased villus height and crypt depth comparing to EN group. Villus height in EN and PN group was 286 ± 65 μm and 180 ± 48 μm (*p* < 0.05), respectively. Crypt depth in EN and PN group was 143 ± 16 μm and 73 ± 24 μm (*p* < 0.05), respectively (Fig. 4B). Additionally, serum FGF19 levels in EN and PN group was 11.0 ± 2.1 ng/ml and 5.6 ± 1.5 ng/ml (*p* < 0.05), respectively (Fig. 4C).

### Effect of FGF19 on the expression of drug metabolism-related genes

**Fig. 5:**
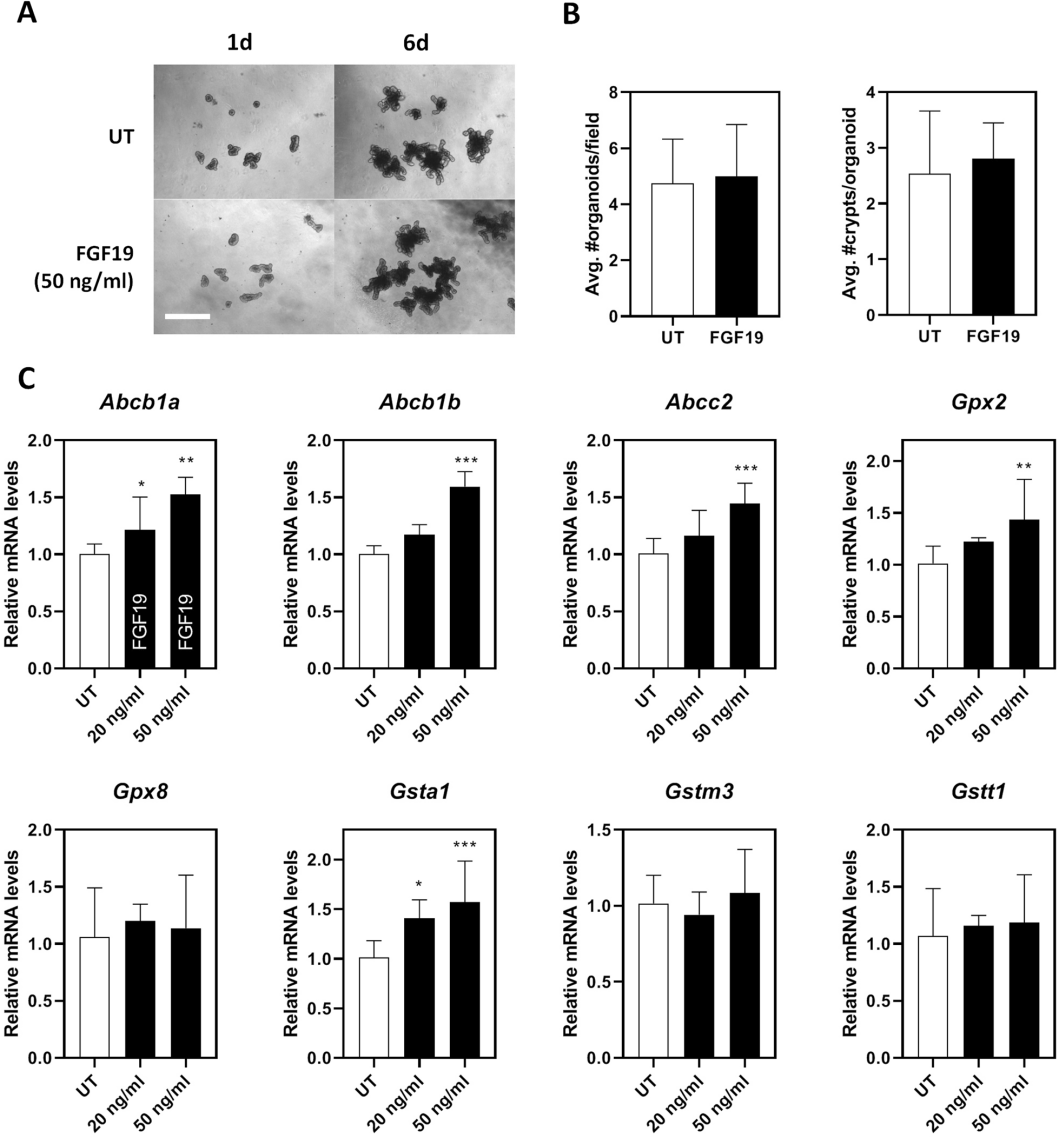
Effect of FGF19 on ABC transporters Glutathione-related genes in ileum organoids. **A** Representative images of organoids treated with FGF19 (50 ng/ml). **B** Expansion and differentiation of organoids described in (A). Data are presented as mean ± SD **C** Relative mRNA levels of ABC transporters and Glutathione-related genes. Data are presented as mean ± SD from three independent experiments. *p < 0.05, **p < 0.01

In order to address whether those changes in drug metabolism-related genes were attributed to reduced levels of FGF19, we then introduced murine ileum organoids as in vitro model. Firstly, we confirmed that FGF19 on the dosage of 50 ng/ml induced no significant impacts on organoid expansion and differentiation. As shown, no significant changes were observed in morphological features (average organoid number/field and average crypt number/organoid) (Fig. 5A, B). Of note, most of those genes changed in vivo were also dramatically regulated by FGF19 in vitro. For ABC transporters, both *Abcb1* (*Abcb1a* and *Abcb1b*) and *Abcc2* were up-regulated by FGF19 treatment in a dose-dependent manner. For glutathione-related genes, *Gpx2* and *Gsta1* were markedly up-regulated by FGF19 treatment, while *Gpx8*, *Gstm3* and *Gstt1* were unchanged. (Fig. 5C). Taken together with above-mentioned in vivo data, these findings suggest changes in Abcb1, Abcc2, Gpx2 and Gsta1 under PN were probably attributed to reduced levels of FGF19.

### Effect of FGF19 on the efflux function of Abcb1

**Fig. 6:**
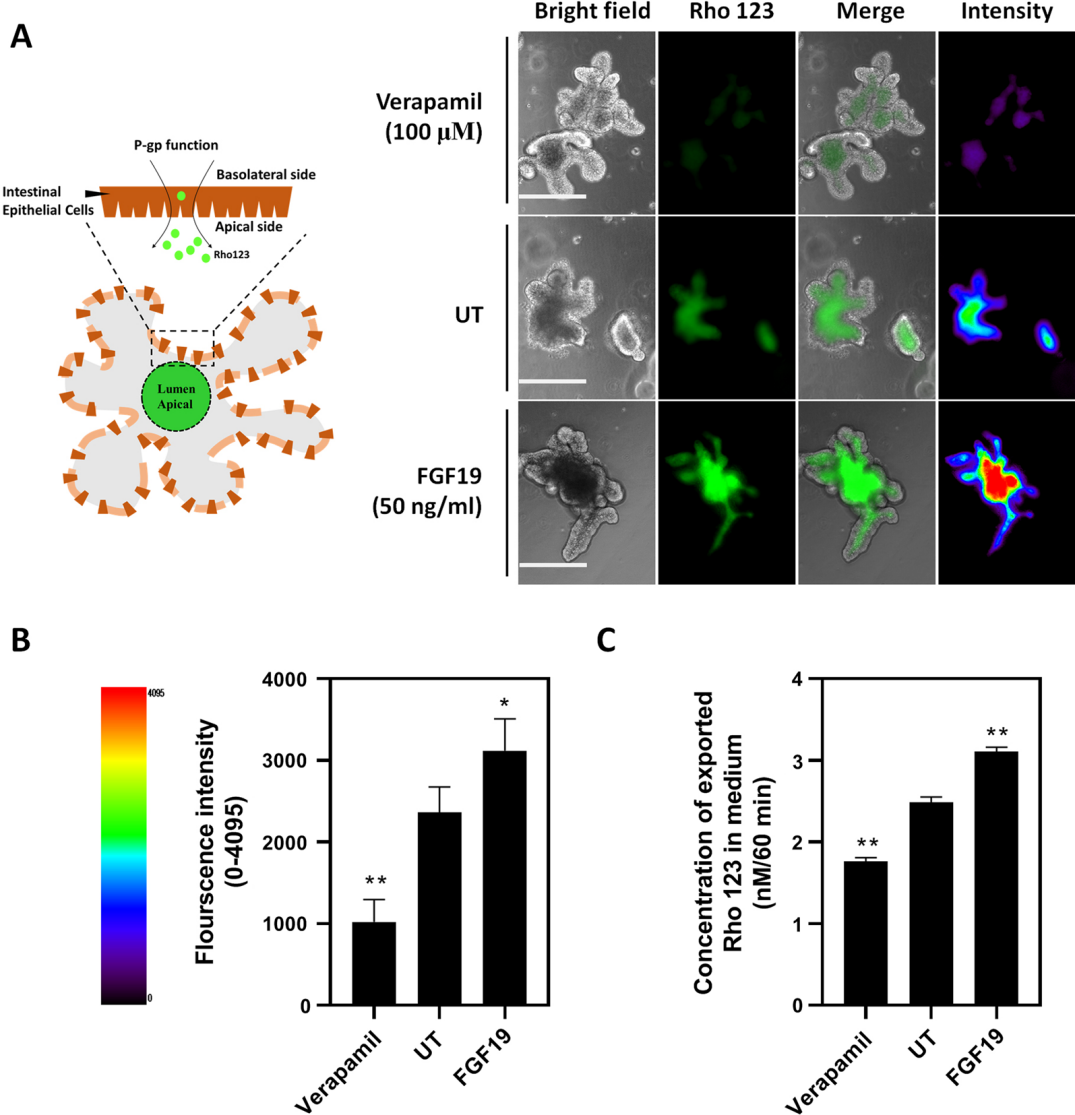
Effect of FGF19 on Abcb1 efflux function. **A** Efflux transport of Rho 123 through Abcb1. Organoids were treated with FGF19 (50 ng/ml) for 6 days. Verapamil (100 uM) served as positive control. Scale bar = 200 μm **B** Fluorescence intensity in organoids described in (**A**). At least 20 organoids were analyzed in each group. Data are presented as mean ± SD. *p < 0.05, **p < 0.01. **C** Rho 123 in medium. Released Rho123 in culture medium was determined as described in Methods section. Data are presented as mean ± SD. **p < 0.01. Three independent experiments were performed that presented similar results

As shown, fluorescence signal of Rho 123 was enriched within the lumen domain of organoids, which was significantly enhanced in FGF19 group, and was suppressed in Verapamil group (Fig. 6A). Quantitatively, average signal intensity in FGF19 group was increased by about 30% compared to UT (untreated) group (*p* < 0.05, Fig. 6B). Consistently, released Rho 123 levels in medium were approximately 2.4 nM/60 min and 3.2 nM/60 min in UT and FGF19 group, respectively (*p* < 0.05, Fig. 6C). Collectively, these results suggested that FGF19 treatment can facilitate the efflux function of Abcb1 in vitro.

## Discussion

According to the United States Healthcare Cost and Utilization Project, it is estimated that over 375,000 patients rely on PN each year in the United States, a number that has almost doubled in the past 20 years [13]. Given that PN mostly serves as a supportive role in nutrient feeding for those patients who are simultaneously receiving oral medication, whether administration of PN can affect drug metabolism raised our great interest. In this study, we introduced piglet model instead of rodent models, because it has more anatomical, physiological, immunological and metabolic similarities with human than any other rodent model [14]. For instance, the pig is more appropriate for modeling liver function and metabolism, as it has hepatic features similar to that of humans and, unlike the rat lack of a gallbladder [15]. The piglet has also been proven to be a useful representation of the human neonate when studying lipid nutrition, including the effect of long-chain n-3 polyunsaturated fatty acids on protein metabolism in the neonate during growth [16]. Overall, the prominence of the piglet in studies concerning human neonatal nutrition reflects the thought that the pig is most similar to humans compared to other animals and therefore the preferred model.

By using ileal tissues, this study represented the first-ever investigation of proteomic profiles in gut to elucidate the whole picture of DEPs by PN treatment. Notably, GO-term analysis revealed a number of DEPs involved in drug binding, namely TOP2A, DHFR, PDE4D, ALB, ATP1B1, DCK (Fig. 1C). Some of these genes are implicated in drug resistance that may significantly affect clinic outcomes. For instance, DHFR (dihydrofolate reductase, 1.45-fold increase in PN) that catalyzes tetrahydrofolate regeneration by reduction of dihydrofolate, is a key factor involved in the resistance of methotrexate [17]; TOP2A (DNA topoisomerase 2a, 1.66-fold increase in PN) that controls and alters the topologic states of DNA during transcription, has been implicated in the resistance of etoposide [18]. Additionally, PPI analysis suggested strong connections among drug binding, RNA recognition and DNA modulation (Fig. 1D), indicating that the delivery of nucleic acid drugs (e.g. anti-viral agents or anti-tumor agents) might be influenced by PN. For instance, more attention should be paid to the use of antibiotics for the management of intestinal infection for pediatric patients receiving PN.

For ABC transporters, we found both Abcb1 and Abcc2 were significantly decreased after PN administration (Fig. 2). The protein product of Abcb1 (P-glycoprotein) is an ATP-dependent drug efflux pump for xenobiotic compounds with broad substrate specificity. Interestingly, anti-viral agents, most of which are nucleic acid drugs that might be influenced by PN as mentioned above, frequently behave as Abcb1 substrates. On the other hand, the protein product of Abcc2 (Mrp2) serves as an efflux pump for some anionic drug conjugates including glucuronides, sulfates and glutathiones, contributing to elimination of a wide range of drugs, including, antihypertensives (e.g., olmesartan and temocaprilate), antineoplastics (e.g., methotrexate, cisplatin, doxorubicin and irinotecan), antiretrovirals (e.g., adefovir, lopinavir and saquinavir), and antibiotics (e.g., ampicillin and azithromycin) [19]. Therefore, we speculate that if the expression of these above-mentioned efflux transporters is decreased in the patients receiving PN, there would be an increase in bioavailability of certain drugs, which would increase drug concentrations in the body leading to potential risks of drug-drug interaction.

On the other hand, excessive endogenous or exogenous toxins may persist in the gut lumen due to gastrointestinal hypomotility under PN, contributing to the development of gut pathologies such as inflammation. Therefore, biotransformation and drug-metabolizing enzymes in gut can be as important as those in liver to metabolic detoxication. In this study, we found that a number of glutathione-related genes like Gpx2 and Gsta1 were decreased in PN group (Fig. 2), as well as the absolute content of GSH (Fig. 3). Gsta1 belongs a member of a family of enzymes that add GSH to target electrophilic compounds, including carcinogens, therapeutic drugs, environmental toxins, and products of oxidative stress [20]; Gpx2 is a member of glutathione peroxidase family which catalyzes the reduction of organic hydro peroxides and hydrogen peroxide by GSH, and thereby protect cells against oxidative damage [21]. GSH is a ubiquitous tripeptide (-glu-cys-gly). Reduced GSH and its oxidized form, GSSG, is the major thiol redox system in cells, of crucial importance for cellular function. In gut, the maintenance of intestinal mucosal GSH status is crucial for detoxication reactions, as sufficient GSH levels can enhance intestinal peroxide metabolism and thus drive peroxide uptake from gut lumen and reduce peroxide output into lymph [22]. Collectively, we speculate that reduction in GSH and GSH-related enzymes by PN may not only impair elimination of cellular ROS, but also facilitate the absorption and distribution of oxidant compounds, toxins or lipophilic drug derivatives via lymph circulation. Nevertheless, despite the absolute content of GSH was obviously reduced, GSH/GSSG ratio was unchanged, suggesting that oxidative injury in gut was less severe than liver, where GSH/GSSG ratio was significantly decreased as described elsewhere [5].

One of the key findings in this study is the role of FGF19 in the dysregulation of these drug metabolism-related genes, a well-studied postprandial hormone involved in the regulation of bile acid homeostasis and energy metabolism. First of all, FGF19 signals can activate FGFR4/β-klotho receptor complex to repress hepatic cholesterol 7α-hydroxylase (CYP7A1), thereby limiting the synthesis of bile acids [23]. In addition, FGF19 can prevent lipid accumulation by inducing fatty acid oxidation and suppressing acetyl coenzyme A carboxylase 2 (ACC2), a key gene involved in fatty acid synthesis [24]. Furthermore, FGF19 can regulate glucose metabolism through stimulation of glycogen synthesis and inhibition of gluconeogenesis via inactivation of cAMP regulatory element-binding protein (CREB) and proliferator-activated receptor g coactivator-1α (PGC-1α) [25]. Therefore, the beneficial impact of FGF19 on glucose, lipid and bile acid homeostasis raise the possibility to pursue FGF19 as a therapeutic target for treating various metabolic disorders including diabetes, non-alcoholic fatty liver disease and cholestatic liver diseases [26, 27]. Given that PN is a condition commonly featured by long-term deprivation of oral feeding along with disrupted bile acid homeostasis, we hypothesized that changes in those above-mentioned genes may be, at least partially attributed to FGF19. For in vivo experiments, we examined serum FGF19 levels in portal vein and morphological changes in ileum where FGF19 is predominantly produced. As expected, PN induced significant villus atrophy leading to reduced production of FGF19 (Fig. 4). For in vitro experiments, we examined the effect of FGF19 on the expression of drug metabolism-related genes by using ileum organoids. One aspect needs comment here. The dosage of FGF19 here was refered to Kim et al.’s work in which the effect of 50 ng/ml of recombinant human FGF19 was clarified on mouse ileum organoids [28]. As FGF19 signaling is also involved in cell survival and proliferation in addition to a wide range of metabolic processes including cholesterol, lipid and glucose, it’s important to discriminate whether these changes in drug metabolism-related genes were resulted from cell proliferation or differentiation. In this study, we found a number of drug-related genes were significantly regulated by FGF19 at 50 ng/ml, including *Abcb1a/b*, *Abcc2*, *Gpx2* and *Gsta1*. Given no morphological changes observed (Fig. 5), we assumed that these changes in the expression of drug metabolism-related genes were attributed to the specific effect of FGF19 on gene regulation, other than non-specific effect on cell survival or proliferation. Finally, we confirmed that the efflux function of Abcb1 was substantially facilitated by FGF19 (Fig. 6), indicating that the regulation of Abcb1 by FGF19 was evident not only on gene levels, but also on protein levels.

Finally, two limitations of this study need comment, 1) the small number of replicates in the proteomic analysis, and 2) no data from other intestinal segments except ileum. Although previous studies have reported impaired intestinal homeostasis under PN, this present study is the first one into abnormal expression pattern of intestinal drug metabolism-related genes. In conclusion, this study indicated that PN can induce significant changes in the expression of drug metabolism-related genes in ileum, which may affect the pharmacokinetc process of certain drugs in clinical practice. Some of the changed genes may be attributed to gut-derived FGF19, including glutathione-related genes (Gsta1 and Gpx2) and ABC transporters (Abcb1 and Abcc2).

## Data Availability

All data generated or analyzed during this study are included in this published article.

## Abbreviations

PN: Parenteral nutrition
EN: Enteral nutrition
DEPs: Differentially expressed proteins
TMT: Tandem mass tag
HCD: High-energy collisional dissociations
qPCR: Quantitative real-time PCR
GO: Gene ontology
PPI: Protein–protein interaction
GSSG: Oxidized glutathione
GSH: Glutathione
FGF19: Fibroblast growth factor 19
ABC transporters: ATP-binding cassette transporters
FXR: Farnesoid x receptor
ROS: Reactive oxygen species
ABCB1: ATP binding cassette subfamily B member 1
ABCC2: ATP binding cassette subfamily C member 2
GST: Glutathione-s-transferase
GPX: Glutathione peroxidase
Rho123: Rhodamine 123
DHFR: Dihydrofolate reductase
TOP2a: DNA topoisomerase 2a
CYP7A1: Cholesterol 7α-hydroxylase
ACC2: Acetyl coenzyme A carboxylase 2
CREB: CAMP regulatory element-binding protein
PGC-1α: Proliferator-activated receptor g coactivator-1α
